# Actin nucleation by WH2 domains at the autophagosome

**DOI:** 10.1038/ncomms8888

**Published:** 2015-07-30

**Authors:** Amanda S. Coutts, Nicholas B. La Thangue

**Affiliations:** 1 Laboratory of Cancer Biology, Department of Oncology, Medical Sciences Division, University of Oxford, Old Road Campus Research Building, Old Road Campus, off Roosevelt Drive, Oxford OX3 7DQ, UK

## Abstract

Autophagy is a catabolic process whereby cytosolic components and organelles are degraded to recycle key cellular materials. It is a constitutive process required for proper tissue homoeostasis but can be rapidly regulated by a variety of stimuli (for example, nutrient starvation and chemotherapeutic agents). JMY is a DNA damage-responsive p53 cofactor and actin nucleator important for cell survival and motility. Here we show that JMY regulates autophagy through its actin nucleation activity. JMY contains an LC3-interacting region, which is necessary to target JMY to the autophagosome where it enhances the autophagy maturation process. In autophagosomes, the integrity of the WH2 domains allows JMY to promote actin nucleation, which is required for efficient autophagosome formation. Thus our results establish a direct role for actin nucleation mediated by WH2 domain proteins that reside at the autophagosome.

## Abstract (web summary)

Autophagy is a catabolic process whereby cellular components are degraded by the autophagosome, but the role of the actin cytoskeleton is not clear. Here Coutts and La Thangue show that the actin nucleator JMY is recruited to the autophagosome via binding LC3, and promotes actin nucleation that is required for autophagosome maturation.

## Graphical abstract

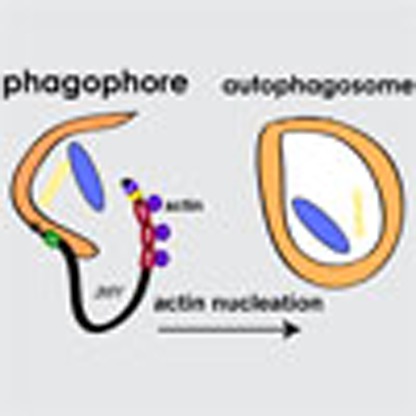

Macroautophagy (referred to as autophagy) is a catabolic process important for cell survival and numerous environmental cues can stimulate autophagy, which is maintained at basal levels to ensure cellular homoeostasis^1^,^2^,^3^. Nutrient deprivation is the most well-studied effector of autophagy, but other types of stress, such as those induced by drugs and chemotherapeutic agents, can trigger autophagy^1^,^4^. Although autophagy can provide a means of tumour cell growth and survival, in some cases it may also be an effective cell death inducer^1^,^4^. When autophagy is initiated, a crescent-shaped double membrane, the isolation membrane or phagophore, is formed that eventually closes in on itself to form the autophagosome around cellular macromolecules, which can include damaged or unwanted organelles or proteins, lipids and nucleic acids^5^,^6^. At later stages of autophagy, the autophagosomes may fuse with endocytic vesicles before ultimately fusing with the lysosome resulting in the degradation of cellular material^6^,^7^. In the initial stages of autophagy, the autophagic proteins are recruited to a membrane to initiate membrane nucleation from a variety of sources including the endoplasmic reticulum, golgi, mitochondria and recycling endosomes^8^.

While studies have identified a large number of Atg (autophagy-related) proteins whose step-wise roles in the autophagy process are becoming clearer, the mechanical processes and proteins (especially non-Atg) involved in regulating the induction, expansion and fusion of the autophagosome are incompletely understood^6^. Although research has demonstrated the involvement of the cytoskeleton, especially the microtubule system, in various aspects of autophagy the role of the actin cytoskeleton in autophagy is unclear^2^. It is interesting to note that while a majority of actin polymerization occurs at the membrane–cytosol interface^9^, in mammalian cells very little is known about the role of actin in autophagy.

JMY was initially described as a cofactor that can influence p53 activity during the DNA damage response^10^,^11^. Since then, JMY has been shown to be a WH2 domain-containing actin nucleator that can shuttle between the nucleus and the cytoplasm, dependent on stress^12^,^13^. Importantly, JMY is unusual in that it can nucleate actin in both an Arp2/3-dependent and -independent fashion, suggesting a highly specialised role^13^.

Here we report that JMY plays a role in cells undergoing autophagy. Significantly, we found that JMY is recruited to LC3-containing autophagosomes when cells are exposed to a variety of autophagy-inducing agents, including starvation and drug treatment. This requires the amino (N)-terminal region of JMY, which contains an LC3-interacting region (LIR), required for JMY to localize at the autophagosome where it enhances autophagosome formation and maturation. Most interestingly, the LIR in JMY is also required for actin nucleation activity, which is necessary for autophagosome formation and maturation. Depletion of JMY leads to markedly decreased cell survival in autophagocytic cells, while JMY overexpression enhances cell survival, dependent on the presence of its actin-nucleating activity and ability to enhance autophagosome formation. Our results establish for the first time actin nucleation at the autophagosome and suggest a mechanistic role for actin in autophagosome formation and maturation.

## Results

### JMY localizes to the autophagosome

**Figure 1:**
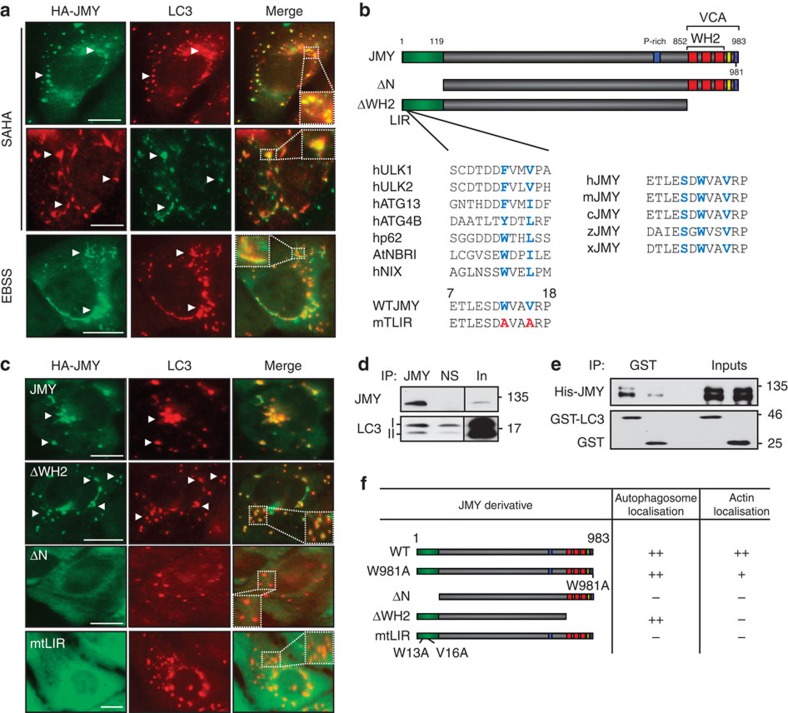
JMY localizes to autophagosomes through an LIR. (**a**) U2OS cells expressing HA-JMY were treated with SAHA or starved with Earle’s balanced salt solution (EBSS) for 6 h. Top panel: HA-JMY was detected with mouse anti-HA and LC3 with rabbit anti-LC3. Middle panel: rabbit anti-HA and mouse anti-LC3 were used. Inset shows enlarged region as denoted. Scale bar, 10 μm. (**b**) Schematic of JMY illustrating the position of the WH2 domains and the central (yellow) and acidic regions (purple), which comprise the VCA region. The proline-rich region (P-rich) preceding the VCA region is denoted in blue. The region removed in ΔN is shown as is the position of the LIR and the tryptophan (W) 981. Numbering refers to amino-acid residue. Lower left: Line-up comparing the JMY LIR region to that of other known LIR-containing proteins. Sequences of some known LIR-containing proteins compared with JMY, adapted from ref. 45. Amino-acid residue position is given. Lower right: The JMY LIR is highly conserved across species. Blue highlighted residues show conservation of core sequence (W/V) and potential phosphorylation site (S). h, human; m, mouse; c, canine; z, zebrafish; x, xenopus; At, *Arabidopsis*. (**c**) JMY and its derivatives were expressed in U2OS cells and treated with SAHA 6 h. JMY was detected with anti-HA antibody and rabbit anti-LC3 was used to detect endogenous LC3. Scale bar, 10 μm. (**d**) MCF7 cells were treated with SAHA before collecting for immunoprecipitation (IP) with anti-JMY antibody (JMY) or non-specific IgG control (ns). Inputs (In) represent 2% of extract. (**e**) His-JMY was incubated with GST-LC3B or GST and complexes isolated using glutathione sepharose 4B. Inputs represent total protein loading. *n*=3 independent experiments. (**f**) Table summarising properties of JMY derivatives. ++=strong,+=moderate, −=no/weak.

As nuclear JMY is responsive to a variety of cellular stressors such as UV and hypoxia^10^,^12^,^14^, and is resident in the cytoplasm where it can influence actin nucleation, we were interested in examining whether JMY takes on a more general role in cellular stress, such as autophagy. In cells treated with a variety of agents that effectively induce autophagy (notably stress induced by drug treatment and nutrient starvation), a proportion of JMY localized to distinct cytoplasmic foci reminiscent of autophagosomes (Supplementary Fig. 1a). During the autophagic process the ATG8 homologue, MAP1LC3 (referred to as LC3), becomes lipidated and cleaved (LC3-II) and is incorporated onto the double-membrane phagophore that is destined to become the autophagosome^5^. Assessing LC3-II either by immunoblotting or visualization of LC3 puncta is the most widely accepted method of determining autophagy levels^15^. A significant proportion of JMY also colocalized with LC3-containing autophagosomes (Fig. 1a and Supplementary Fig. 1b), indicating that JMY can accumulate at autophagosomes during autophagy induction.

Inhibiting autophagic flux can be accomplished by preventing turnover of LC3-II containing autophagosomes using either tubastatin A or bafilomycin A1 (refs 16, 17). These treatments resulted in the accumulation of LC3 foci and colocalization of JMY with LC3 (Supplementary Fig. 1b). Autophagy targets macromolecules for degradation but JMY levels were not consistently downregulated during autophagy (Supplementary Fig. 1c,d). The autophagy cargo protein p62 (sequestosome 1/SQSTM1) interacts with LC3 via an LIR and is known to be degraded during autophagy^18^ and, as expected, its levels are consistently upregulated during bafilomycin A1 (a late phase autophagy inhibitor^19^) treatment in contrast to JMY (Supplementary Fig. 1e).

### JMY contains an LIR

To identify the region involved in targeting JMY to the autophagosome, we expressed JMY derivatives (Fig. 1b) in drug-treated (SAHA) U2OS cells to induce autophagy. Previously, JMY was shown to be capable of inducing actin-containing structures in cells, via its WH2 domain-containing VCA region^12^,^13^ (Fig. 1b). The C-terminal region containing the WH2 domains was dispensable for the localization of JMY at the autophagosome (Fig. 1c and Supplementary Fig. 2a). Surprisingly, removal of the N-terminal region of JMY (Fig. 1b) completely ablated the ability of JMY to colocalize with LC3 at the autophagosome (Fig. 1c and Supplementary Fig. 2a). Closer examination of this region revealed a sequence with similarity to an LIR (Fig. 1b). In general, the LIR confers on proteins the ability to interact with members of the LC3 family and target to the phagophore, and is present in proteins recruited as components of the autophagy machinery, including ATG1 (ULK1/2), ATG13 and ATG4 (Fig. 1b)^20^. The LIR sequence resembles the consensus W/F/YxxL/I/V (Fig. 1b), frequently in the presence of surrounding acidic residues with a serine (S) or threonine (T)^21^. Of note, JMY has a number of acidic residues flanking the motif as well as an adjacent S residue (Fig. 1b). The JMY LIR is highly conserved across species, arguing for its importance in JMY function (Fig. 1b). Mutating the critical tryptophan (W) and valine (V) to an alanine (A) in JMY (mtLIR, Fig. 1b) resulted in a complete loss of autophagosome localization (Fig. 1c and Supplementary Fig. 2b). Indeed, rather than localizing to autophagosomes, mtLIR remained evenly distributed throughout the cell (Fig. 1c and Supplementary Fig. 2b). Moreover, JMY and LC3 interact in cells as LC3 co-immunoprecipitated with JMY (Fig. 1d and Supplementary Fig. 2c). Importantly, JMY could interact directly with LC3B (Fig. 1e), which was mediated by the N-terminal half of JMY (Supplementary Fig. 2d). Further, the interaction between the N-terminal region of JMY and LC3 was dependent on the presence of an intact LIR (Supplementary Fig. 2e). To provide further evidence that the LIR in JMY is a functional LIR, we swapped the LIR in p62 with the LIR in JMY (Supplementary Fig. 2f), which maintained the localization of p62 at the autophagosome during both SAHA and starvation-induced autophagy (Supplementary Fig. 2f). Thus, JMY contains a functional LIR required for targeting its activity to the autophagosome.

### JMY influences autophagosome formation

**Figure 2:**
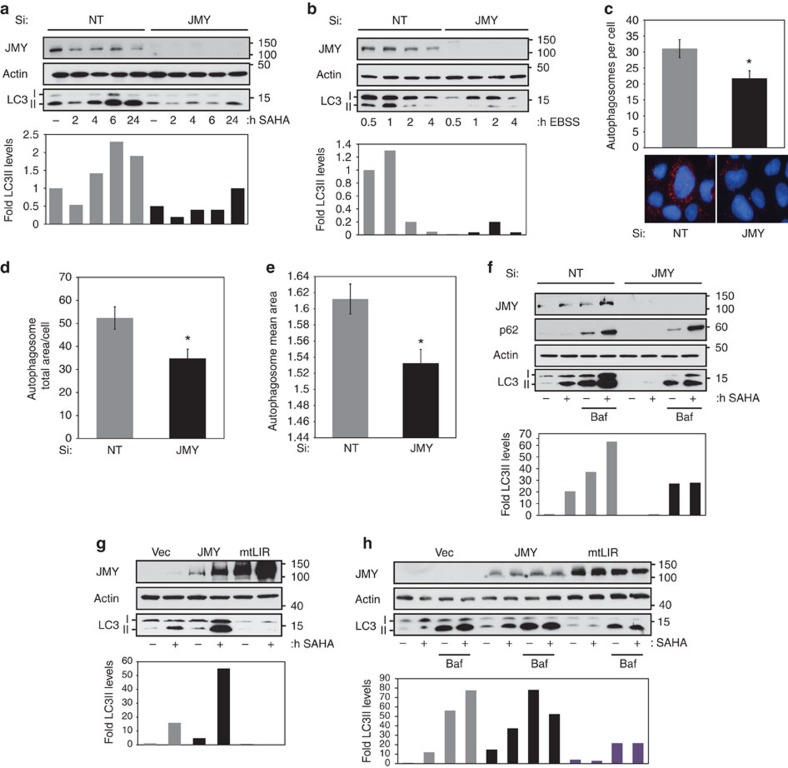
JMY influences autophagosome formation. (**a**) U2OS cells were treated with non-targeting (NT) or JMY siRNA for 72 h before treating with SAHA for the indicated time points. Actin was used as a loading control. *n*=8 independent experiments. The graph below shows fold LC3-II levels after normalizing for actin. (**b**) U2OS cells were treated with NT or JMY siRNA for 72 h before replacing the medium with EBSS for the indicated time points. *n*=5 independent experiments. The graph below shows fold LC3-II levels after normalizing for actin. (**c**) Quantitation of autophagosome number in U2OS cells treated with NT or JMY siRNA for 72 h. Cells were treated with SAHA for 6 h before fixation and processing for immunofluorescence. Graph represents the number of autophagosomes/cell calculated using an IN Cell Analyser 1000. The data represent *n*=3 independent experiments with >3,000 cells per treatment. *P<0.02, Student’s *t*-test. (**d**) Quantitation of total autophagosome area/cell from cells treated as in **c**. *P<0.005, Student’s *t*-test. (**e**) Quantitation of mean autophagosome area from cells treated as in **c**. *P<0.002, Student’s *t*-test. (**f**) U2OS cells were treated with NT or JMY siRNA for 72 h. The last 24 h cells were treated with vehicle (−) or SAHA (+) with or without bafilomycin A1 (Baf) for 4 h. Actin was used as a loading control. *n*=4 independent experiments. The graph below shows fold LC3-II levels after normalizing for actin. (**g**) U2OS cells stably expressing HA-JMY (JMY), HA-JMYmtLIR (mtLIR) or vector (vec) control were treated with or without SAHA for 24 h. *n*=4 independent experiments. The graph below shows fold LC3-II levels after normalizing for actin. (**h**) U2OS cells stably expressing HA-JMY (JMY), HA-JMYmtLIR (mtLIR) or vector (vec) control were treated with SAHA with or without bafilomycin A1 (Baf) as indicated. *n*=3 independent experiments. The graph below shows fold LC3-II levels after normalizing for actin. Error bars in all graphs are mean±s.e.m.

We next explored the possibility that JMY influences autophagosome formation and might thus be involved in the maturation process. Time-course studies demonstrated that depletion of JMY resulted in a marked reduction in LC3-II levels during drug-induced autophagy (Fig. 2a and Supplementary Fig. 3a,b). This was unlikely to be an off-target effect as three individual small interfering RNAs (siRNAs) targeting JMY resulted in a similar decrease in LC3-II levels (Supplementary Fig. 3c). Similar results were also seen during starvation-induced autophagy (Fig. 2b and Supplementary Fig. 3d,e). Of note, JMY depletion was able to significantly impede the formation of LC3-II during short time periods of stress treatment (Fig. 2a,b and Supplementary Fig. 3a–e), suggesting that JMY acts at an early stage in autophagosome formation. We next examined the effect of JMY depletion on the number of autophagosomes in individual cells by immunofluorescence, where we observed a significant decrease in the number of autophagosomes per cell during drug-induced autophagy (Fig. 2c). This reduction in autophagosome number was also reflected in reduced total autophagosome area per cell (Fig. 2d). Interestingly, we also observed a reduction in the mean autophagosome area in JMY-depleted cells (Fig. 2e), suggesting that JMY influences autophagosome maturation.

As a decrease in LC3-II levels could be due to decreased formation or increased turnover, we used bafilomycin A1 (which inhibits the late phase of autophagy by preventing fusion with the lysosomes^19^) to uncouple these processes. Notably, JMY depletion did not prevent autophagic flux, as treatment with bafilomycin A1 was able to enhance LC3-II levels even in the presence of JMY siRNA treatment, but JMY-depleted cells still demonstrated lower LC3-II levels (Fig. 2f and Supplementary Fig. 3f), consistent with a defective autophagy process. In addition, autophagy involves fusion of the phagosome with the lysosome^15^, but we were unable to observe any significant colocalization of JMY with lysosomal markers (cathepsin D and LAMP2; Supplementary Fig. 3g,h), further supporting the notion that JMY is involved in a stage of autophagy that is prior to lysosomal fusion.

We created stable cell lines with wild-type JMY and the mtLIR derivative to explore the role of the LIR motif in the ability of JMY to influence autophagosome development. Wild-type JMY-expressing cells had an increased level of LC3-II compared with control cells (Fig. 2g and Supplementary Fig. 2g,h), supporting the earlier conclusion that JMY has a role in autophagosome formation. Interestingly, cells stably expressing mtLIR demonstrated consistently reduced levels of LC3-II when compared with wild-type-expressing cells (Fig. 2g and Supplementary Fig. 2g,h). Indeed, in mtLIR expressing cells LC3-II levels often appeared reduced compared with their control-treated counterparts (Fig. 2g and Supplementary Fig. 2g,h), yet were able to undergo autophagic flux (as assessed using bafilomycin A1 treatment; Fig. 2h). Overall, these results indicate that JMY is involved in autophagosome formation and highlight the importance of the LIR motif in this role.

### Actin nucleation is involved in autophagosome formation

Interestingly, the N-terminal region of JMY is required for its ability to form cytoplasmic actin-containing structures, despite the presence of the WH2 domains (Fig. 1f and Supplementary Fig. 4a). More surprisingly, mutation of the LIR alone was sufficient to prevent the formation of the majority of cytoplasmic actin structures by JMY (Supplementary Fig. 4a), even though we were still able to observe colocalization of JMY and actin at the leading edge (Supplementary Fig. 4a), where previously it has been shown to localize to influence cell motility^12^,^13^. This suggested to us a potential link between actin nucleation and autophagosome formation. Therefore, we tested whether JMY was able to induce actin-containing structures during autophagy, and examined the colocalization of JMY and actin under conditions of autophagy. Notably, we observed significant colocalization of JMY with cytoplasmic actin structures, but not with mtLIR (Supplementary Fig. 4b,c).

**Figure 3:**
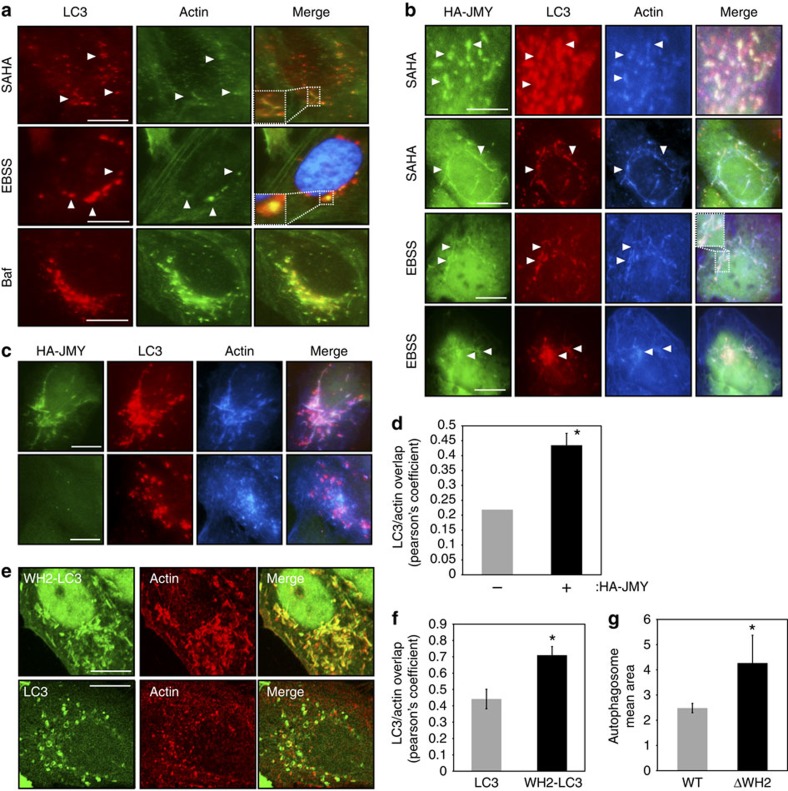
Actin is involved in autophagosome formation. (**a**) U2OS cells treated with SAHA, bafilomycin A1 (Baf) or starved with EBSS, all for 4 h. Rabbit anti-LC3 and mouse-actin antibodies were used. Insets show enlarged regions. Scale bar, 10 μm. (**b**,**c**) U2OS cells expressing JMY and derivatives were treated for 6 h with SAHA or EBSS as denoted. JMY was detected with goat anti-JMY antibody L16, actin with mouse anti-actin and LC3 with rabbit anti-LC3. Scale bar, 10 μm. (**d**). Quantitative data of cells treated as in **c**. Data are represented as the Pearson’s coefficient of the LC3 and actin signal correlation in the presence or absence of JMY. *n*=>20 cells per treatment, two independent experiments. *P<0.0001, Student’s *t*-test. (**e**) U2OS cells expressing mcherry–GFP–LC3 (LC3) or mcherry–GFP–WH2–LC3 (WH2–LC3) were treated with SAHA (6 h). Actin was visualized with mouse anti-actin antibody and false coloured red for visualization purposes. Scale bar, 10 μm. (**f**) Quantitative data of cells treated in **e**. Data are represented as the Pearson’s coefficient of the LC3 and actin signal correlation. *n*=>20 cells per treatment, two independent experiments. *P<0.001, Student’s *t*-test. (**g**) The mean area of the autophagosomes quantified in 6 h SAHA treated HA-JMY and HA-JMYΔWH2 expressing U2OS cells using an IN Cell Analyser 1000, *n*=>150 cells per treatment, two independent experiments. *P<0.1, Student’s *t*-test. Error bars in all graphs are mean±s.e.m.

Since the ability of JMY to induce actin-containing cytoplasmic structures requires not just the WH2 domains but also an intact LIR (Fig. 1f), we reasoned that actin may be involved in autophagosome development during which JMY takes on a mechanical role. This idea was compatible with the fact that JMY expression induced elongated autophagosomes, reminiscent of cytoplasmic JMY-induced actin-containing structures observed in normal conditions (compare inset Supplementary Fig. 4a with Supplementary Fig. 4d). As JMY can incorporate globular (G)-actin into actin-containing structures in cells^12^,^13^, we performed *in situ* actin incorporation assays under conditions when JMY is present at autophagosomes. Indeed, incorporation of actin at the sites of JMY localization was apparent (Supplementary Fig. 4e). Significantly, during both drug treatment and starvation-induced autophagy, we observed colocalisation of actin with LC3 at autophagosomes (Fig. 3a). Occasionally these structures had an elongated filamentous structure similar to those observed with JMY (Fig. 3a top panel, enlarged inset). Moreover treatment with bafilomycin A1 resulted in some cells displaying a striking colocalization of LC3 with actin-containing foci (Fig. 3a lower panel). Most importantly, we observed colocalization of JMY and actin with LC3 at autophagosomes (Fig. 3b), and further the presence of JMY enhanced the localization of actin at the autophagosome (Fig. 3c,d), arguing that JMY is rate-limiting for the presence of actin at autophagosomes.

Although the ability of JMY to localize at autophagosomes does not require its WH2 domains (Fig. 1c and Supplementary Fig. 2a), we reasoned that JMY may influence autophagosome formation through its actin-nucleating activity, which is targeted to the autophagosome dependent on an intact LIR (Fig. 1c and Supplementary Fig. 4a–c). To explore this possibility we fused the WH2 domain region of JMY to the N-terminus of LC3 in the context of mcherry–GFP–LC3 (Supplementary Fig. 5a; ref. 22), where JMY would not interfere with lipidation and membrane association of LC3 which occurs at the C terminus^23^. The addition of the WH2 domain region of JMY enhanced the level of actin colocalization with the autophagosomes when compared with mcherry–GFP–LC3 (Fig. 3e,f and Supplementary Fig. 5b,c). We also often observed markedly elongated autophagosomes in mcherry-GFP-WH2-LC3-expressing cells which colocalized with actin (Fig. 3e), again arguing that JMY-dependent actin nucleation at the autophagosome influences autophagosome formation. We noted that autophagosomes formed in the presence of JMYΔWH2 were frequently enlarged and globular (Supplementary Fig. 5d), which was reflected in an increased mean area of the autophagosomes (Fig. 3g). Moreover, the autophagosomes in JMYΔWH2 cells often retained a prominent perinuclear location (Supplementary Fig. 5d). Together these observations suggest that aberrant development of the autophagosome occurs in the absence of actin nucleation.

**Figure 4:**
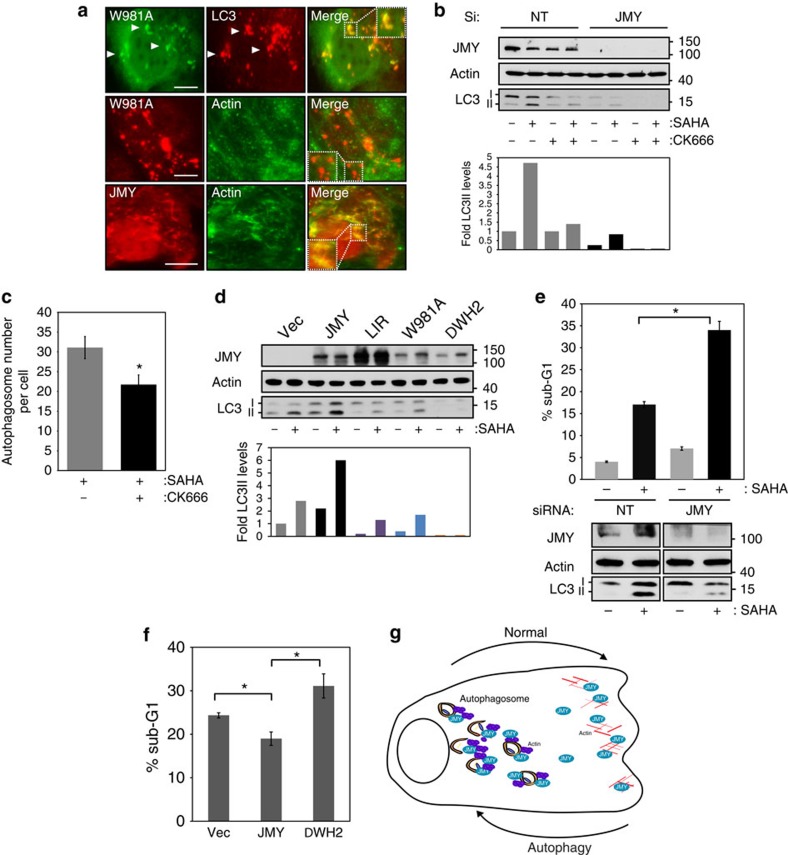
JMY influences survival during autophagy. (**a**) U2OS cells expressing HA-JMY and derivatives were treated with SAHA for 6 h before fixation. Scale bar, 10 μm. (**b**) U2OS cells were treated with control (NT) or JMY siRNA for 72 h. Six hours before collecting cells were treated with SAHA with or without the Arp2/3 inhibitor (CK-666; 20 μM) as denoted before collecting. The graph below shows fold LC3-II levels after normalizing for actin. (**c**) Quantitation of autophagosome number from cells treated with SAHA with or without CK-666 for 6 h. Graph represents *n*=3 independent experiments with >1,200 cells per treatment. *P<0.02, Student’s *t*-test. (**d**) U2OS cells stably expressing HA-JMY and derivatives were treated with or without SAHA for 6 h before collecting. The graph below shows fold LC3-II levels after normalizing for actin. (**e**) U2OS cells were treated with control (NT) or JMY siRNA for 72 h. The cells were treated with vehicle control (−) or SAHA for 24 h before collecting and analysed by FACS. Graph represents percentage sub-G1 of a representative experiment. *n*=4 independent experiments, *P<0.02, Student’s *t*-test. Blots underneath represent input protein levels. (**f**) U2OS cells stably expressing HA-JMY and derivatives were treated with SAHA for 24 h before collecting and analysed by FACS. Graph represents percentage sub-G1 of a representative experiment. *n*=3 independent experiments, *P<0.05 Student’s *t*-test. (**g**) Model depicts the cytoplasmic role of JMY in influencing autophagosome formation through its actin nucleation activity. Under normal conditions cytoplasmic JMY is able to induce the formation of new actin filaments to facilitate cell motility and invasion. During conditions of stress leading to autophagy induction JMY is localized at the autophagosome where it fosters autophagosome formation via its actin-nucleating activity, leading ultimately to enhanced cell survival. Actin is depicted by both red lines (leading edge) and purple circles (autophagosome) to reflect differences in localization rather than chemical or structural composition. All error bars are mean±s.e.m.

JMY can nucleate actin in both an Arp2/3-dependent and -independent fashion 12,13 and mutation of a tryptophan (W981A) residue in the WH2 domain region prevents Arp2/3-dependent actin nucleation^13^. Although loss of Arp2/3-dependent actin nucleation did not alter the ability of JMY to locate to autophagosomes (Fig. 4a) it compromised, rather than abolished, the ability to colocalize with actin during autophagy (Fig. 4a compare middle and lower panels). This suggested that Arp2/3-dependent actin nucleation plays a role in autophagosome formation. To explore this idea further, we treated cells with the Arp2/3 inhibitors CK-666 and CK-869 (ref. 24) during autophagy which resulted in a reduction in LC3-II levels and the number of autophagosomes, suggesting a dependency on Arp2/3 activity for autophagosome formation (Fig. 4b,c and Supplementary Fig. 6a–c). Further, using a panel of JMY stable cell lines we compared the effects of actin nucleation on LC3-II levels during autophagy. While loss of Arp2/3-dependent actin nucleation in W981A rendered JMY less effective at inducing autophagy compared with wild-type JMY, it was not completely compromised (Fig. 4d). On the other hand, removal of the entire WH2 domain resulted in a more dramatic reduction in LC3-II (Fig. 4d). Together these observations suggest that JMY is able to influence autophagosome formation in both an Arp2/3-dependent and -independent fashion by having a mechanical role dependent on actin nucleation.

### JMY impacts on cell survival during autophagy

Autophagy can both promote and inhibit the growth of cancer cells^1^. As drug treatment, for example, the HDAC inhibitor SAHA, is a potent inducer of apoptosis as well as autophagy^25^, we depleted JMY from cells treated with SAHA and performed FACS analysis to examine the effects. Interestingly, JMY depletion resulted in a significant enhancement of apoptosis (Fig. 4e). We also observed similar effects on apoptosis driven by mTOR inhibition (Supplementary Fig. 6d), suggesting that JMY acts as a pro-survival factor by facilitating autophagy. Moreover, under starvation conditions JMY was able to augment viability (Supplementary Fig. 6e,f).

Numerous studies have implied a role for p53 in autophagy regulation^26^,^27^,^28^ and, previously, we have shown that JMY is a p53 cofactor capable of influencing p53-dependent cell survival^10^,^11^,^12^. To explore the possibility of a transcriptional role of JMY in LC3 regulation, we examined LC3 mRNA levels in JMY-depleted cells at time points during autophagy where JMY influences L3C-II levels. JMY depletion caused a modest decrease in LC3 mRNA levels under starvation (Supplementary Fig. 7b), but not following 6 h SAHA treatment (Supplementary Fig. 7a). To further ascertain if the effects of JMY could be mediated in part by its nuclear role as a p53 cofactor, we depleted JMY in matched p53^+/+^ and p53^−/−^ HCT116 cells. Regardless of the presence or absence of p53, JMY depletion still resulted in a decrease in LC3-II levels (Supplementary Fig. 7c), moreover, JMY-induced PARP cleavage was not dependent on p53 (Supplementary Fig. 7c). Thus, the influence of JMY on cell survival during autophagy is mediated primarily through a cytoplasmic role of JMY independent of p53 activity. Indeed, the ability of JMY to influence cell survival correlated with actin nucleation activity and modulation of LC3 levels, as JMYΔWH2 cells exhibited higher levels of apoptosis compared to wild-type JMY expressing cells (Fig. 4f). Together these results suggest that JMY, through its influence on autophagosome formation in an actin-mediated fashion, is able to influence cell viability (Fig. 4g).

## Discussion

Autophagy is highly relevant for numerous diseases including cancer, lysosomal storage diseases, neurodegenerative diseases as well as processes such as aging, development and immunity^29^. Our study has demonstrated a novel role for JMY in autophagy, where it acts to promote autophagosome formation through a functional LIR motif. In a similar fashion to JMY, other LIR motif-containing proteins contribute to various stages of autophagosome formation and maturation^21^. However, the role of JMY in autophagy appears novel and attributable to its ability to nucleate actin. Thus, JMY augments the level of actin present in the autophagosome, and the integrity of its WH2 domain region coincides with efficient autophagosome formation. The fact that the autophagosomes formed in the presence of a JMY derivative lacking actin-nucleating activity are often enlarged suggests defects in autophagosome processing and maturation, likely dependent on actin nucleation. Of interest, the JMYmtLIR lacks most cytoplasmic actin colocalization suggesting that a functional LIR is required to allow actin nucleation via the WH2 domains in the C-terminal region. This would be consistent with a mode of regulating JMY’s activity via allosteric mechanisms and autoinhibition, similar to other WH2 domain-containing proteins such as WASP^30^. Moreover, the fact that JMYmtLIR is still able to colocalize with cortical actin filaments suggests that there is a separation of JMY’s ability to nucleate actin to influence autophagosome formation and that involved in directing cell motility events.

JMY belongs to a family of type I actin nucleation-promoting factors, which include WHAMM, WASH and WASP^31^. These nucleation-promoting factors have a common C-terminal region that contains the WH2 domain-containing VCA region and a poly-proline-rich region but diverge at their N-terminus^31^,^32^. WHAMM that shares the highest degree of identity to JMY has been shown to be involved in endoplasmic reticulum to Golgi transport^33^, while mammalian WASH localizes to early and recycling endosomes^34^,^35^. WASH has been shown to be a negative regulator of autophagy, although the influence of its actin-nucleating activity on this ability is unknown^36^,^37^. It is likely that JMY and other actin-nucleating proteins have distinct cellular roles based on their localization to a particular organelle and in this regard the divergent N-terminal region may provide specificity, as well as control, over actin-nucleating activity. The fact that JMY’s expression appears to be restricted to higher eukaryotes^38^,^39^ suggests that it may function in a more specialized form of autophagy required in higher organisms. This would allow JMY to function specifically at the autophagosome where the N-terminal region is required not only for targeting but also activation of the actin-nucleating activity in the C-terminus. Previous work has hinted at a role for actin in autophagsome formation in mammalian cells, although the underlying mechanism is unknown^17^,^40^ and our data support a role for actin nucleation provided by JMY in this process.

JMY is thus a multifunctional protein that combines its actin-nucleating activity with a functional LIR to nucleate actin and foster autophagosome maturation and thereby impact on cell survival (Fig. 4g). In particular JMY’s ability to influence the outcome of cellular stress may be two-fold; in the cytoplasm it can act to promote autophagy leading to survival, while in the nucleus JMY can enhance p53-dependent apoptosis^10^,^12^. Autophagy is highly relevant for numerous diseases including cancer, lysosomal storage diseases, neurodegenerative diseases, as well as processes such as aging, development and immunity^29^. Our study provides a link between actin nucleation and autophagy with cell survival during stress. This, in turn, may also provide novel opportunities to modulate autophagosome levels that will have a significant impact on cell survival.

## Methods

### Plasmids, antibodies and reagents

The following plasmids have been previously described; pcDNA3 HA-JMY, JMYΔN, ΔWH2, pET28a JMY^11^,^12^. HA-p62 was a gift from Qing Zhong^41^ (Addgene plasmid #28027). JMYmtLIR and p62/JMY LIR were generated using the Stratagene QuickChange Site-directed mutagenesis kit. mCherry–GFP–LC3 was a generous gift from Terje Johansen, University of Tromso, Norway^22^. mcherry-GFP-WH2-LC3 was made by fusing the WH2 region of JMY (amino acids 842-982) N-terminal to the LC3 coding sequence of mcherry–GFP–LC3. All constructs were verified by sequencing. Rabbit anti-HA Y-11 (sc-805), goat anti-JMY L16 (sc-10027), mouse anti-p53 DO-1 (sc-126) and mouse anti-p62 (SQSTM1; sc-28359) were from Santa Cruz. Mouse anti-HA antibody HA11 was from BAbCO. Mouse anti-actin (A1978) and anti-tubulin (T6199) antibodies were from Sigma. Mouse anti-LC3 (0231-100/LC3-5F10) antibody was from Nanotools and rabbit anti-LC3B (#2775) and cleaved-PARP (#9541) antibodies were from Cell Signaling. Mouse anti-JMY antibody has been previously described^42^. Primary antibodies for western blotting were used at 1/1,000 with the exception of actin (1/100,000), tubulin (1/10,000) and JMY (hybridoma supernatant 1/250 dilution). Uncropped scans are provided in Supplementary Fig. 8. Primary antibodies for immunofluorescence were used at 1/200 dilution. HRP-conjugated secondary antibody was from DAKO (1/10,000 dilution). Alexa Fluor conjugated secondary antibodies and Alexa Fluor 488 conjugated actin were from Molecular Probes (1/500 dilution). SAHA (Selleckchem) was used at a concentration of 10 μM, rapamycin (Sigma) 1 μM, AZD2014 (Selleckchem) 15 μM, tubastatin A (ChemieTek) 10 μM and bafilomycin A1 (Sigma) 100 nM, unless noted otherwise. Phalloidin-TRITC was from Sigma. The Arp2/3 inhibitors CK-666 and CK-869 were from Merck Biosciences.

### Cell lines and generation of stable cell lines

HeLa, MCF7, HCT116 p53^+/+^, HCT116 p53^−/−^ and U2OS cells were grown in 5% FCS-DMEM plus antibiotics under 5% CO_2_. HeLa, MCF7 and U2OS cell lines were from Sigma (ECACC) and paired HCT116 cell lines were a kind gift from B. Vogelstein (Howard Hughes Medical Institute, The Johns Hopkins University, MD, USA). U2OS cells stably expressing JMY constructs were obtained after transfection of the appropriate construct into U2OS cells and carrying out selection with 500 μg ml^−1^ G418.

### Transfection

Plasmid transfections were performed using GeneJuice (Merck Biosciences). siRNA transfections were performed using Oligofectamine (Invitrogen). In all cell types 25 nM siRNA was used. Human JMY siRNA has been previously described in ref. 12 (JMY1: 5′-gcaacuagaaagcaucaaa-3′; JMY2: 5′-cacucggauugaagaugaa-3′; JMY3: 5′-ccaucacacaguacaacua-3′) and control non-targeting control siRNA #3 was from Dharmacon.

### Immunostaining and quantification

Cells were seeded onto 13 mm glass coverslips, and fixed with 3.7% formaldehyde followed by ice cold methanol for 10 min. Permeabilisation of the cells was performed for 5 min with 0.5% Triton X-100 in phosphate-buffered saline (PBS) followed by incubation with primary antibody for an hour at room temperature (or overnight). Coverslips were washed with 0.025% Tween in PBS extensively before adding secondary antibody. Coverslips were mounted on microscope slides using Vectashield with or without DAPI (4,6-diamino-2-phenylindole; to visualize nuclei) as required. Images were obtained using either an Olympus BX51 inverted fluorescence or Zeiss LSM 780 confocal microscope using a 63 × oil-immersion lense. Autophagosome numbers, mean and total area were calculated using the IN Cell Analyser 1,000 (GE Healthcare). LC3/actin overlap was calculated using Fiji/Image J^43^.

### Immunoprecipitations

For endogenous interactions cells were harvested in TNN buffer (150 mM NaCl, 50 mM Tris-HCl pH 7.4, 5 mM EDTA, 0.5% NP40 in the presence of protease inhibitors) after incubating with 10 μM SAHA for 6 h. Lysates were precleared with protein A/G slurry for 30 min at 4 °C. Immunoprecipitations were performed with goat anti-JMY antibody L16 in the presence of protein A/G slurry overnight at 4 °C.

### *In vitro* binding assays

His-tagged JMY was purified from BL21 cells under native conditions using Ni-NTA agarose according to the QIAexpressionist protocol (Qiagen). Approximately 1 μg of His-tagged JMY was incubated with 1 μg of commercially prepared GST-LC3B (Enzo Life Sciences) or GST control for 1 h in the presence of TNN buffer before adding glutathione sepharose 4B for 30 min at 4 °C. *In vitro* transcribed and translated proteins were obtained using the TnT-coupled reticulocyte lysate system (Promega) in the presence of T7 polymerase according to manufacturer’s instructions. TnT (10 μl) lysate was incubated with 1 μg of GST-LC3B protein in the presence of TNN buffer for 1 h at 4 °C before adding glutathione sepharose 4B for 30 min at 4 °C to immunoprecipitate complexes.

### G-actin incorporation assay

*In situ* G-actin incorporation assays were performed as previously described^44^ using 0.4 μM Alexa Fluor 488 labelled G-actin. Cells grown on coverslips were treated for 2 min at room temperature before fixation with 0.4 μM alexa conjugated G-actin together with 1 mM ATP in permeabilization buffer (0.2 mg ml^−1^ saponin, 20 mM Hepes pH 7.4, 138 mM KCl, 4 mM MgCl_2_ and 3 mM EGTA). Cells were subsequently fixed and processed for immunofluorescence as described under immunostaining.

### FACS analysis

Cells were seeded into 6 cm dishes and treated as appropriate before harvesting. Growth media were collected and adherent cells were lifted by adding 1 ml of trypsin per dish. The cells were pelleted (800 × *g*) for 5 min at 4 °C and washed once with PBS. The cells were fixed in ice cold 70% ethanol/PBS (v/v). Fixed cells were washed with PBS and stained in 2% (v/v) propidium iodide in the presence of 125 U ml^−1^ DNAse-free RNAse A. Stained cells were analysed using flow cytometry (Accuri C6, BD Bioscience).

### MTT assays

MTT assays were performed in 96-well plates using an initial cell number of 2,000 cells per well and all treatments were performed in triplicate. Cells were plated in 100 μl of complete medium and allowed to adhere overnight. The following day the medium was carefully removed and replaced with either complete medium or EBSS and cells were left for 24 h. MTT activity was measured by treating cells with 10 μl MTT reagent (thiazolyl blue tetrazolium bromide; 5 mg ml^−1^ in PBS) per well and incubating at 37 °C for 2 h. The medium was carefully removed and 100 μl dimethylsulfoxide was added to each well and incubated at room temperature for 10 min with agitation. Absorbance was measured at 570 nm with a reference reading at 650 nm.

### RNA isolation, reverse transcription and qPCR

RNA was isolated using the ReliaPrep miniprep system (Promega) according to manufacturer’s instructions. RNA (2 μg) was reverse transcribed using MMLV and oligodT primers before performing real-time PCR using Brilliant III Ultra-Fast SYBR QPCR MM (Agilent Technologies). GAPDH was used as an internal control and human LC3B primer sequences were from Scherz-Shouva *et al*.^28^. Primer sequences were as follows: GAPDH F: 5′-ttcattgacctcaactacat-3′; R: 5′-gtggcagtgatggcatggac-3′; LC3B F: 5′-accatgccgtcggagaag-3′; R: 5′-atcgttctattatcaccgggatttt-3′. Results were calculated using 2^−ΔΔCt^.

## Additional information

**How to cite this article**: Coutts, A. S. *et al*. Actin nucleation by WH2 domains at the autophagosome. *Nat. Commun.* **6**:7888 doi: 10.1038/ncomms8888 (2015).

## Supplementary Material

Supplementary Information Supplementary Figures 1-8
