# Supplementary material for: Actin nucleation by WH2 domains at the autophagosome
Source: Nat Commun. 2015 Jul 30;6:7888. doi: 10.1038/ncomms8888 (PMC4532831; doi:10.1038/ncomms8888)

Supplementary information

Supplementary Figures

Supplementary Figure 1

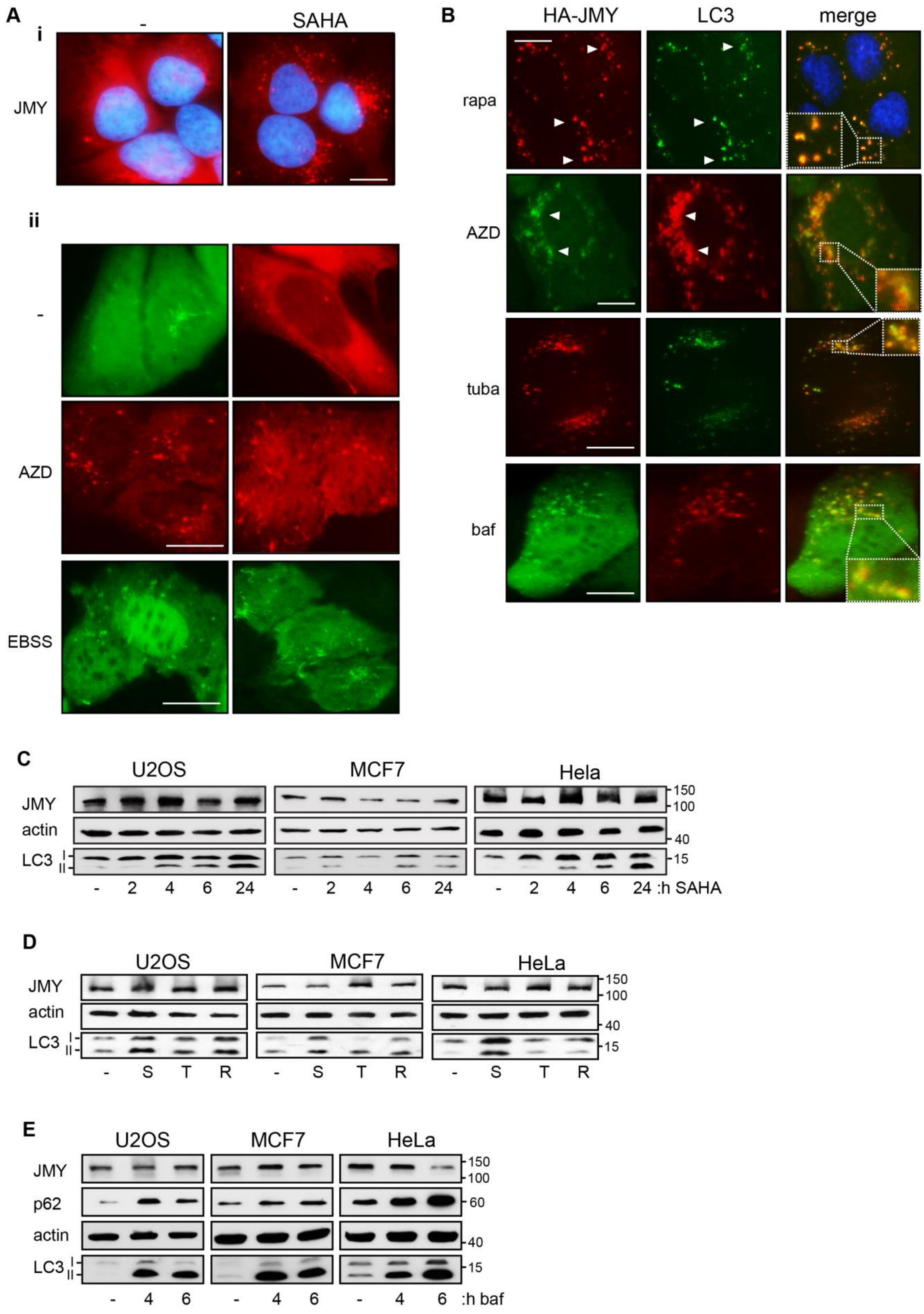

Supplementary Figure 1. A. i. HA-JMY expressing U2OS cells were treated with SAHA (6h). DAPI was used to visualise nuclei. **ii.** U2OS cells stably expressing HA-JMY were untreated (-) or treated with AZD2014 (AZD) or grown under starvation conditions (EBSS) for 6h. JMY was detected with anti-HA. Scale bar = 10 μm. **B.** U2OS cells expressing HA-JMY were treated with rapamycin (rapa, overnight), AZD2014 (AZD, 4h) tubastatin A (tuba; overnight) or bafilomycin A1 (baf; 4h). Inset shows enlarged region as denoted. JMY was detected with anti-HA. LC3 was detected using mouse (green) or rabbit (red) anti-LC3. Scale bar = 10 μm. **C**. Endogenous JMY levels in various cell lines after treating with SAHA as denoted. Actin was used as a loading control. **D**. Endogenous JMY levels in various cell lines after treating overnight with SAHA (S), rapamycin (R) or tubastatin A (T). **E.** Endogenous JMY levels in various cell lines treated with bafilomycin A1 (baf) for the time points indicated.

Supplementary Figure 2

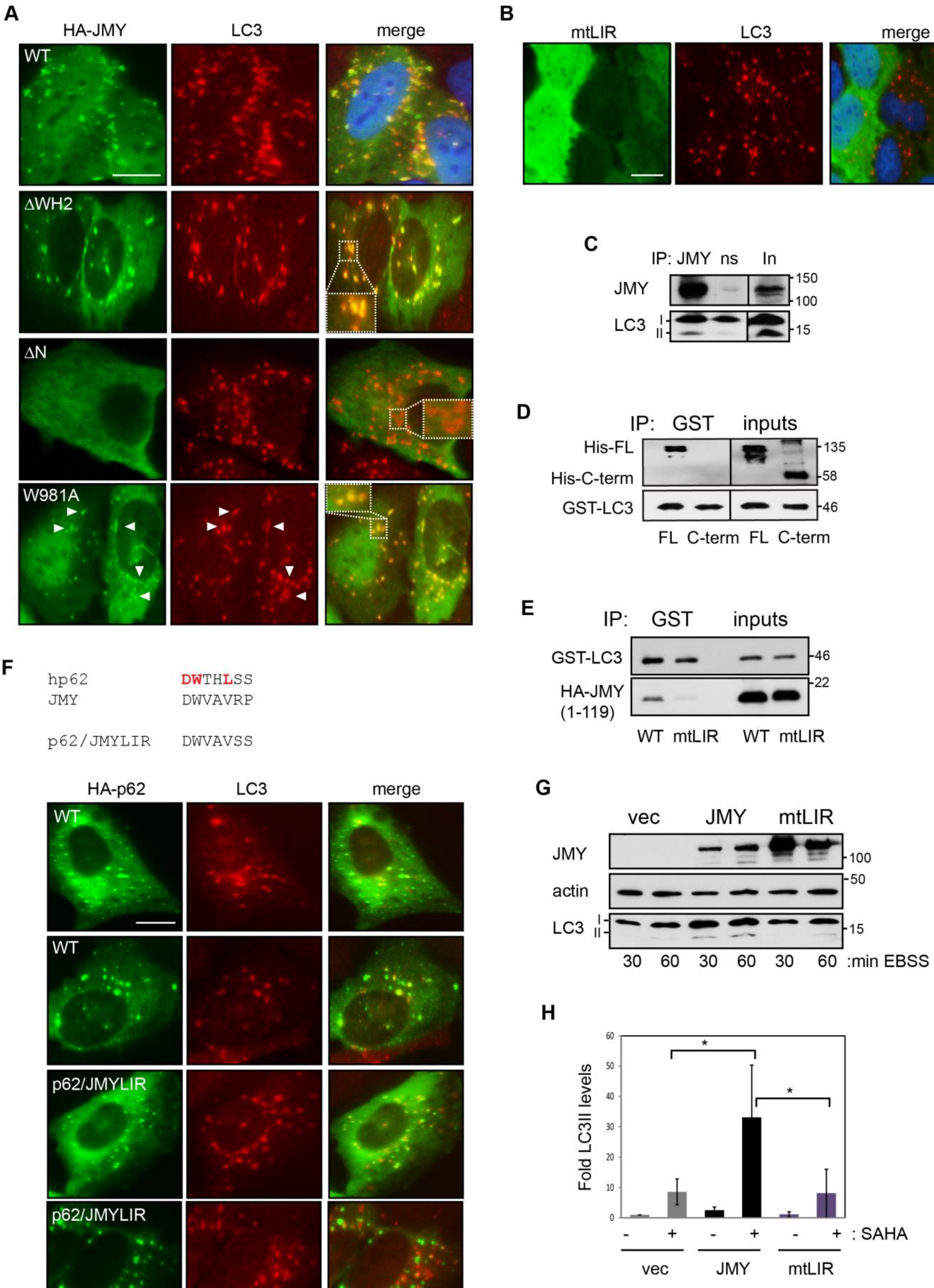

Supplementary Figure 2. A. U2OS cells expressing JMY and derivatives were grown in the presence of EBSS for 6h. JMY was detected with anti-HA and rabbit anti-LC3 was used to detect LC3. Scale bar = 10 μm. **B.** U2OS cells stably expressing JMYmtLIR (mtLIR) were grown in EBSS for 6h. LIR was detected with anti-HA and LC3 with anti-LC3B. Scale bar = 10 μm. **C.** Hela cells were treated with SAHA before harvesting for IP with anti-JMY antibody (JMY) or non-specific IgG control (ns). Inputs (In) represent 2% of extract. n=2 independent experiments. **D.** His-JMY (FL) or C-terminal JMY (C-term; 502-983) was incubated with GST-LC3B and complexes isolated using glutathione sepharose 4B. Inputs represent 50% of total. n=2 independent experiments. Samples were run on the same gel but non-contiguous. **E.** *In vitro* transcribed and translated HA-JMY N-terminal region (1-119; WT) or mtLIR proteins (as denoted) were incubated with 1 μg of GST-LC3B before immunoprecipitating with glutathione sepharose beads. Inputs represent 25% of total. **F.** U2OS cells expressing HA-p62 with wild-type LIR (WT) or JMY LIR (p62/JMYLIR) sequences were grown in the presence of SAHA (6h) or EBSS (2h). p62 was detected using anti-HA antibody and LC3 with rabbit-anti-LC3. Scale bar = 10 μm. Residues in p62 LIR shown to make direct contact with LC3 [21] are highlighted in red. **G.** U2OS cells stably expressing HA-JMY (JMY), HA-JMYmtLIR (mtLIR) or vector (vec) control were grown in EBSS for the time points indicated before harvesting. Actin was used as a loading control. **H.** U2OS cells stably expressing HA-JMY (JMY), HA-JMYmtLIR (mtLIR) or vector (vec) control were treated with or without SAHA for 24h. Graph represents n=4 independent experiments, *p<0.08, Student's t-test.

Supplementary Figure 3

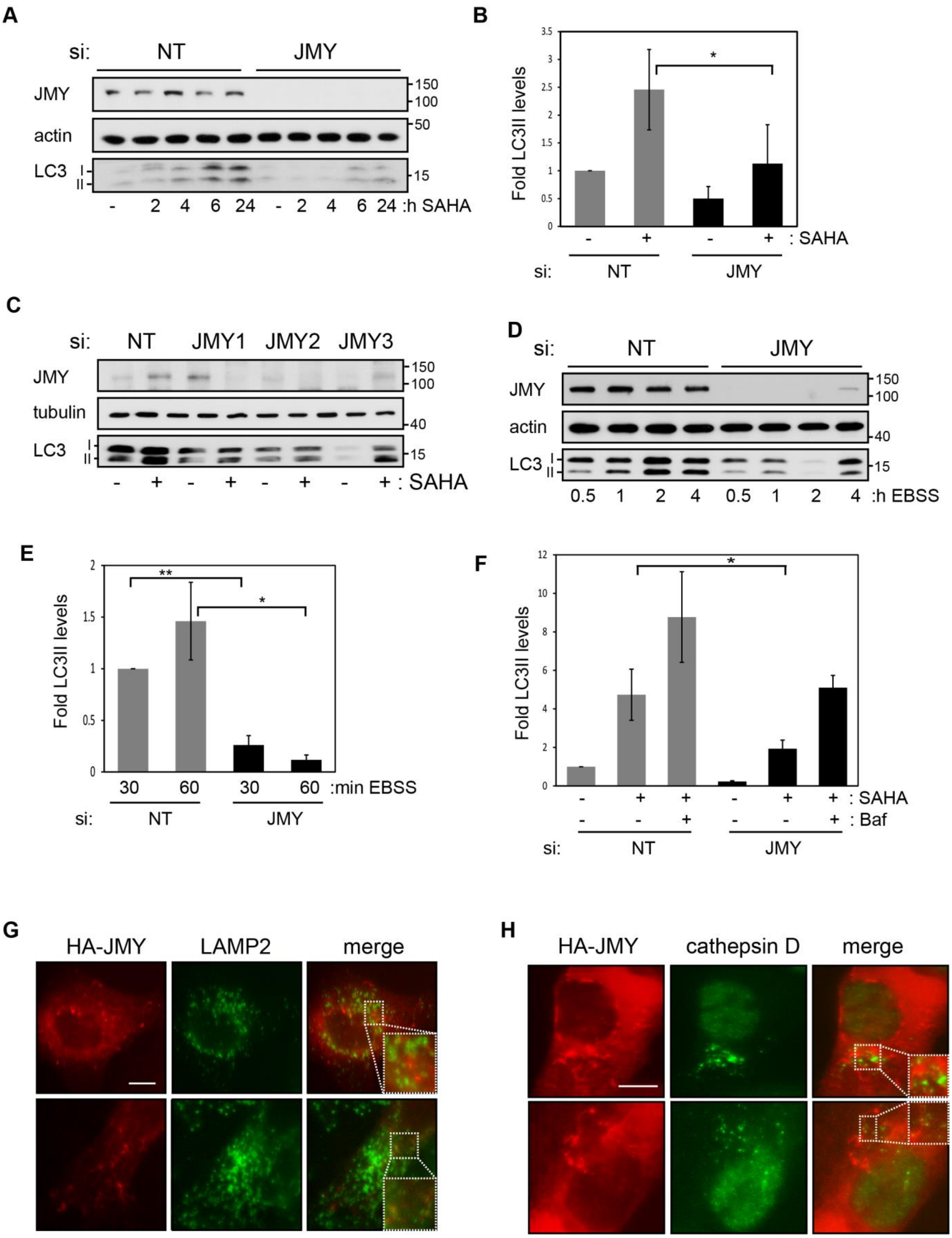

Supplementary Figure 3. A. MCF7 cells were treated with non-targeting (NT) or JMY siRNA for 72h before SAHA treatment for the times indicated. **B.** U2OS cells were treated with non-targeting (NT) or JMY siRNA for 72h before treating with SAHA for the indicated

time points. Actin was used as a loading control. Graph represents n=6 independent experiments, *p <0.05, Student's t-test. **C.** U2OS cells were treated with non-targeting (NT) or JMY siRNAs 1-3 for 72h. Six hours before harvesting, cells were treated with SAHA (+) or vehicle control (-). Actin was used as a loading control. n=3 independent experiments. **D.** HeLa cells were treated with non-targeting (NT) or JMY siRNA for 72h before replacing with EBSS for the indicated time points. **E.** U2OS cells were treated with non-targeting (NT) or JMY siRNA for 72h before replacing the medium with EBSS for the indicated time points. Graph represents n=5 independent experiments, *p<0.05, **p<0.001, Student's t-test. **F.** U2OS cells were treated with non-targeting (NT) or JMY siRNA for 72h. The last 24h cells with treated with vehicle (-) or SAHA (+) with or without bafilomycin A1 (baf) for 4h. Actin was used as a loading control. Graph represents n=3 independent experiments, *p=0.05. **G.** U2OS cells stably overexpressing HA-JMY were treated with SAHA for 6h. JMY was detected with rabbit anti-HA and mouse anti-LAMP2 was used to detect endogenous LAMP2. **H.** U2OS cells stably overexpressing HA-JMY were treated with SAHA for 6h. Rabbit anti-HA was used to detect JMY and mouse anti-cathepsin D was used to detect endogenous cathepsin D.

Supplementary Figure 4

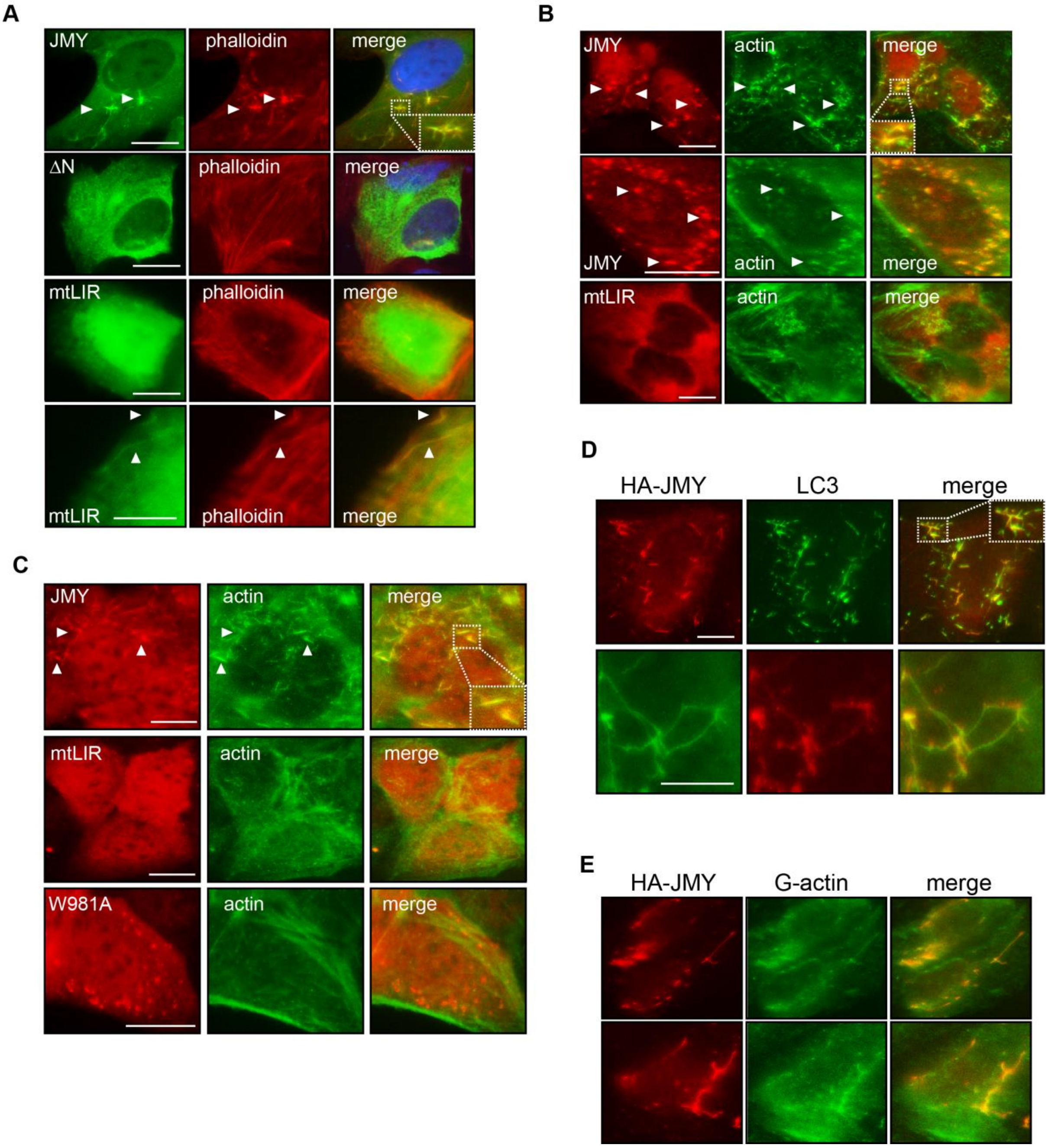

Supplementary Figure 4. **A.** U2OS cells expressing HA-JMY and derivatives. JMY was detected with anti-HA antibody and phalloidin was used to visualise F-actin. Arrows denote areas of JMY and phalloidin colocalisation. Inset denotes enlarged region. Scale bar = 10 μm. **B.** U2OS cells expressing HA-JMY and HA-JMYmtLIR (mtLIR) were treated with SAHA for 6h. JMY was detected with rabbit anti-HA antibody and actin was detected with mouse anti-actin antibody. Inset denotes enlarged region. Scale bar = 10 μm. **C.** U2OS cells

expressing HA-JMY and derivatives were grown in EBSS for 6h. JMY was detected with rabbit anti-HA antibody and actin was detected with mouse anti-actin antibody. Inset denotes enlarged region. Scale bar = 10 μm. **D.** U2OS cells expressing HA-JMY were treated with SAHA for 6h. JMY was detected with anti-HA antibody and anti-LC3 was used to visualise autophagosomes. Inset denotes enlarged region. Scale bar = 10 μm. **E.** U2OS cells stably expressing HA-JMY grown on coverslips before performing G-actin-incorporation assays using 0.4μM AlexaFluor 488-labelled G-actin. Assays were carried out for 2 min at room temperature before fixation and processing for IF. JMY was detected with anti-HA antibody.

Supplementary Figure 5

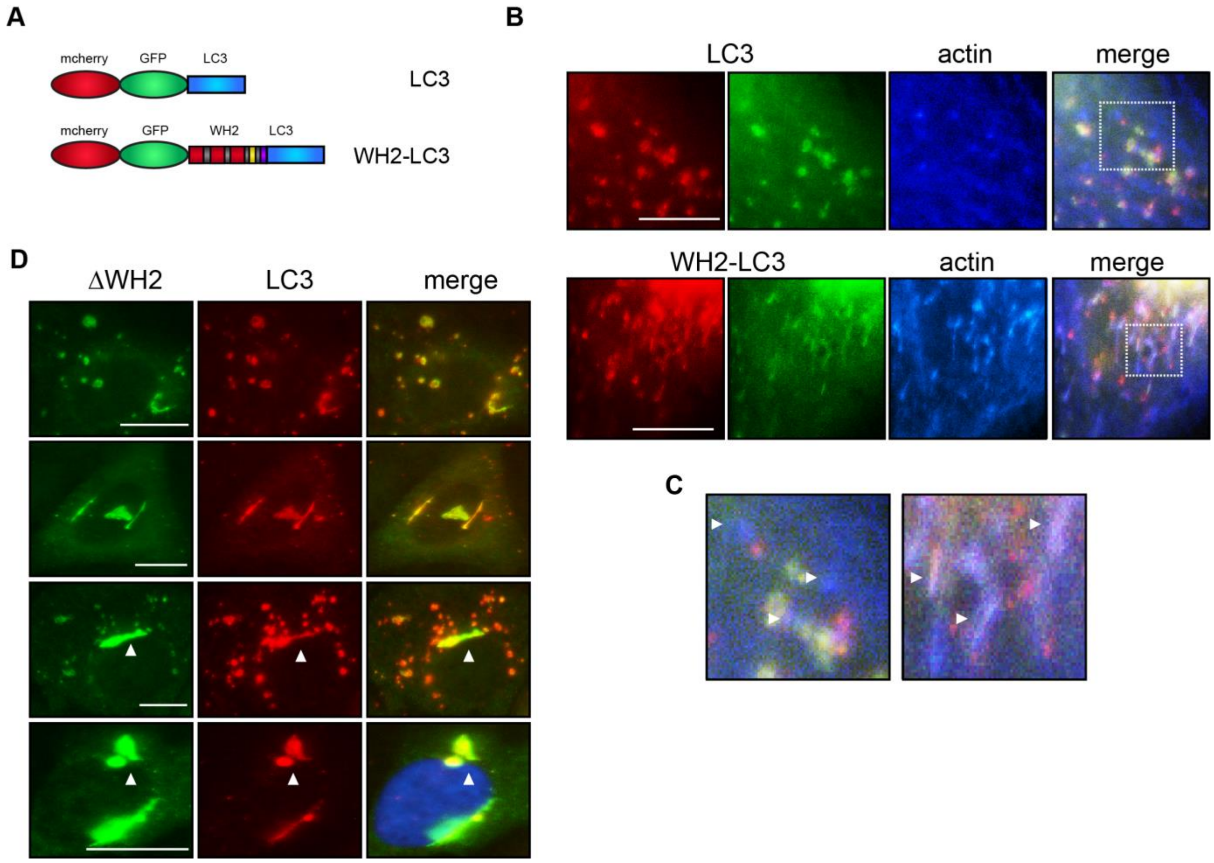

Supplementary Figure 5. A. Schematic illustrates the mcherry-GFP-LC3 (LC3) and mcherry-GFP-WH2-LC3 (WH2-LC3) constructs. **B.** U2OS cells expressing mcherry-GFP-LC3 or mcherry-GFP-WH2-LC3 were treated with rapamycin A. Actin was visualised with mouse-anti-actin antibody. **C.** Enlarged area of mcherry-GFP-LC3 (left) compared to mcherry-GFP-WH2-LC3 (right) as denoted by hatched boxes in **B**. Arrows denote actin foci that do not colocalise with mcherry-GFP-LC3 (left) compared to the significant colocalisation of actin with mcherry-GFP-WH2-LC3 (arrows, right). Scale bar = 10 μm. **D.** U2OS cells expressing HA-JMYΔWH2 were treated with SAHA 6h before processing for IF. Anti-HA and anti-LC3 antibodies were used. Arrows denote enlarged perinuclear autophagosomes. DAPI was used to visualise nuclei. Scale bar = 10 μm.

Supplementary Figure 6

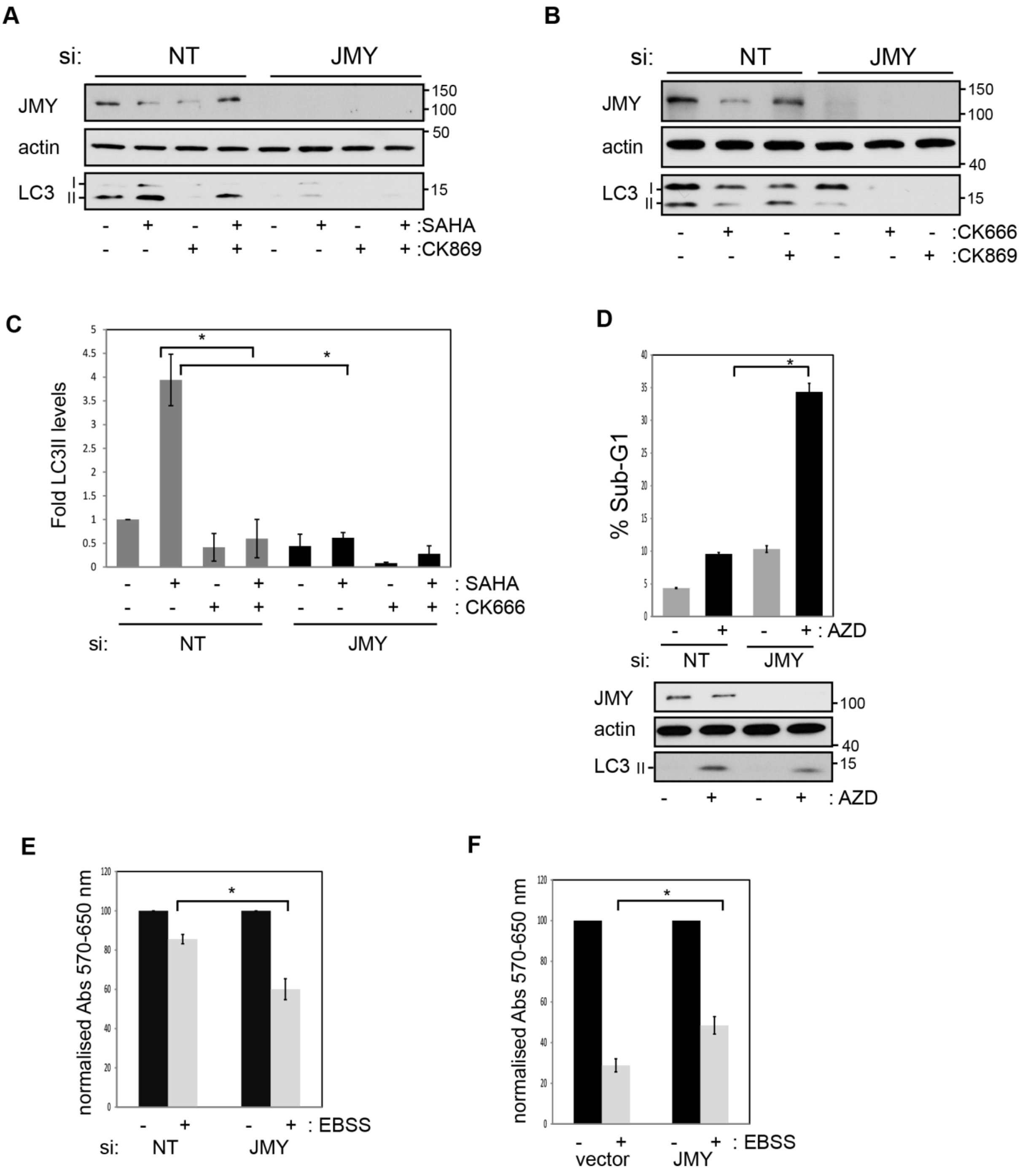

Supplementary Figure 6. A. U2OS cells were treated with control (NT) or JMY siRNA for 72h. Six hours before harvesting cells were treated with SAHA, with or without Arp2/3 inhibitor (CK-869; 20 μM) before harvesting. **B.** U2OS cells were treated with control (NT) or JMY siRNA for 72h. Two hours before harvesting the medium was replaced with EBSS with or without Arp2/3 inhibitor (CK-666 or CK-869; 40 μM) as denoted before harvesting. **C.**

U2OS cells were treated with control (NT) or JMY siRNA for 72h. Six hours before harvesting cells were treated with SAHA with or without the Arp2/3 inhibitor (CK-666; 20 μM) as denoted before harvesting. Graph represents n=3 independent experiments, *p<0.05, Student's t-test. **D.** U2OS cells were treated with control (NT) or JMY siRNA for 72h. The cells were treated with vehicle control (-) or AZD2014 for 24h before harvesting and analysed by FACS. Graph represents percentage sub-G1 of a representative experiment. n=3 independent experiments, *p<0.001, Student's t-test. Blots underneath represent input protein levels. **E.** MTT assays were performed with U2OS cells treated with control (NT) or JMY siRNA for 72h before replating in DMEM (-) or EBSS (+) for 24h. Graph represents mean +/- s.e.m with DMEM (control) values normalised to 100% and EBSS values expressed as a percentage of control. Representative experiment is shown, n=2 independent experiments. *p<0.005, Student's t-test. **F.** MTT assays were performed with U2OS cells stably expressing HA-JMY (JMY) or vector. Cells were grown for 24h in DMEM (-) or EBSS (+) before performing MTT assay. Graph represents mean +/- s.e.m with DMEM (control) values normalised to 100% and EBSS values expressed as a percentage of control. Representative experiment is shown, n=2 independent experiments. *p=0.01, Student's t-test.

Supplementary Figure 7

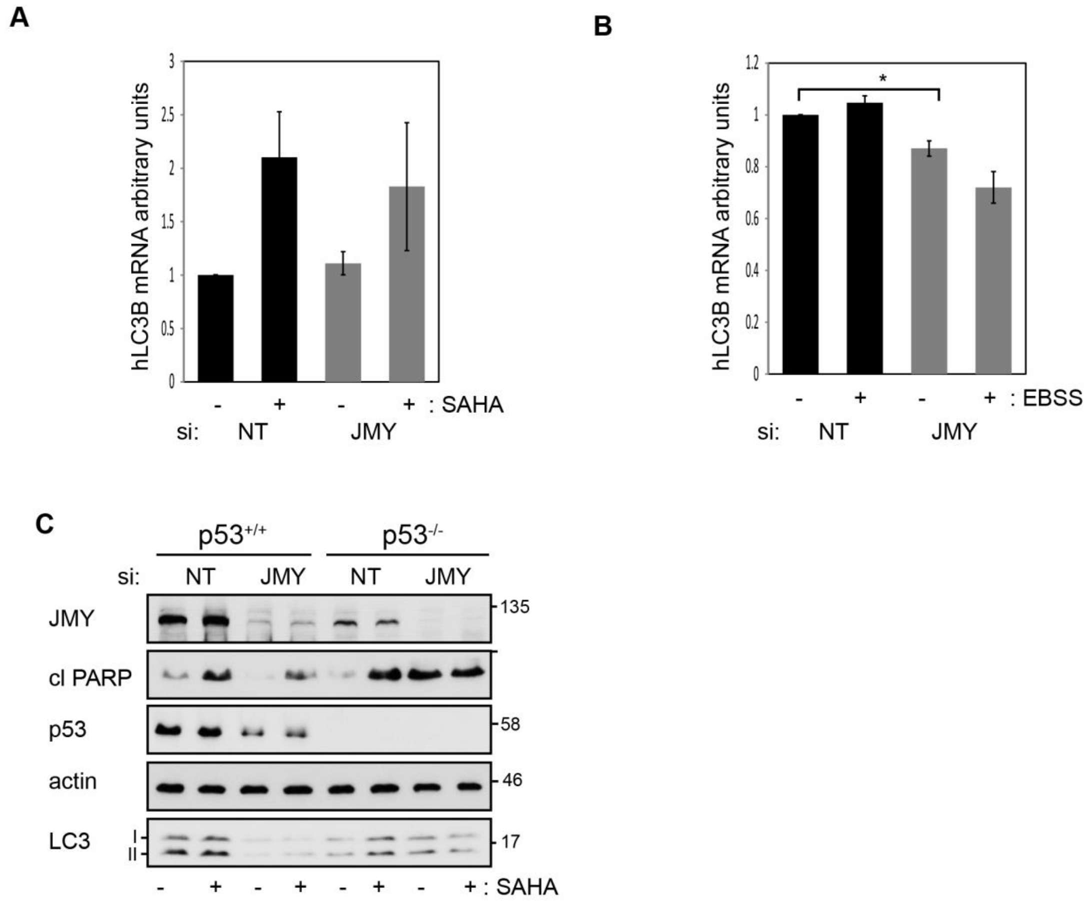

Supplementary Figure 7. A. qPCR analysis of hLC3B mRNA levels from U2OS cells treated with either vehicle (-) or 6h SAHA (+). Values were normalised to GAPDH and expressed as fold over control (NT) untreated. n=4 independent experiments. **B.** qRT-PCR analysis of hLC3B mRNA levels from U2OS cells grown in either DMEM (-) or EBSS for 30 min (+). Values were normalised to GAPDH and expressed as fold over control (NT) untreated. n=3 independent experiments. *p<0.05, Student's t-test. **C.** HCT116 $p53^{+/+}$ or $p53^{-/-}$ cells were treated with control (NT) or JMY siRNA for 72h. Six hours prior to harvesting cells were treated with SAHA (+) or vehicle (-). A representative experiment is shown, n=3 independent experiments.

Supplementary Figure 8 uncropped blots for all primary data in Figures 1-4

Figure 1C: endogenous IP

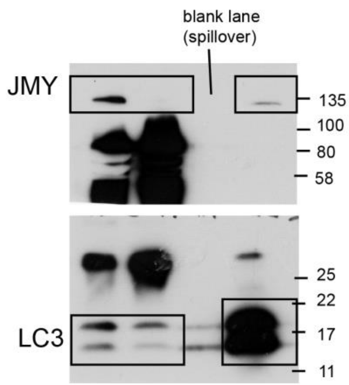

Figure 1E: in vitro IP

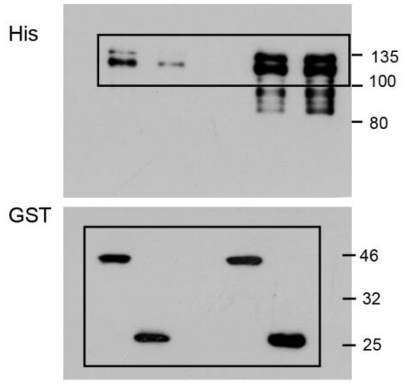

Figure 2A: JMY siRNA effects on LC3

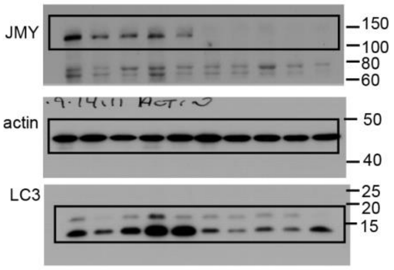

Figure 2B: JMY siRNA effects on LC3

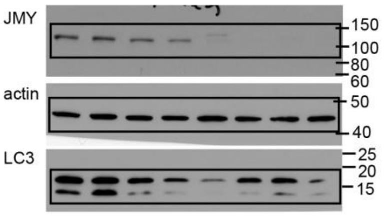

Figure 2F: Baf effects: actin section probed 1st with actin followed by p62

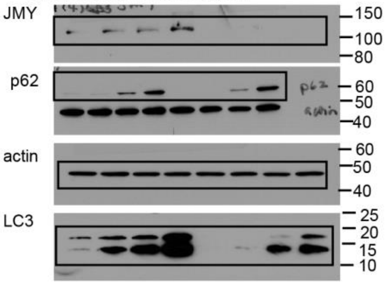

Figure 2G: Stable cell line WT vs LIR

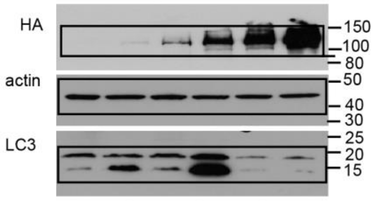

Figure 2H: Stable cell line WT vs LIR with Baf

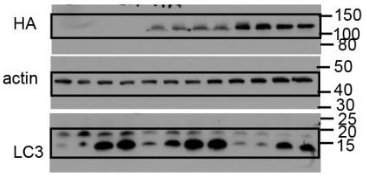

Figure 4B: Effect of Arp2/3 inhibitor

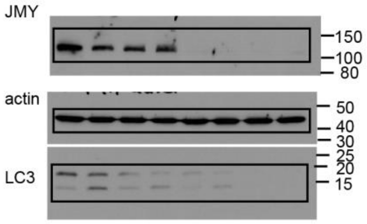

Figure 4D: Stable cell lines LC3 levels

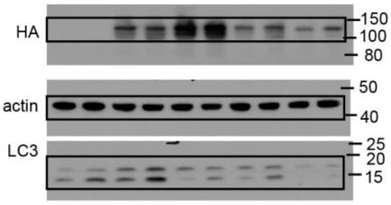

Figure 4E: FACS inputs

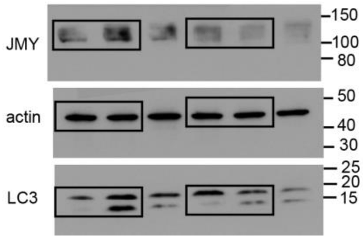

Supplement: Supplementary Information — Supplementary Figures 1-8 [file ncomms8888-s1.pdf]
